# Effect of perioperative bronchodilator therapy on postoperative pulmonary function among lung cancer patients with COPD

**DOI:** 10.1038/s41598-021-86791-1

**Published:** 2021-04-16

**Authors:** Sun Hye Shin, Sumin Shin, Yunjoo Im, Genehee Lee, Byeong-Ho Jeong, Kyungjong Lee, Sang-Won Um, Hojoong Kim, O. Jung Kwon, Jong Ho Cho, Hong Kwan Kim, Yong Soo Choi, Jhingook Kim, Jae Ill Zo, Young Mog Shim, Juhee Cho, Danbee Kang, Hye Yun Park

**Affiliations:** 1 Division of Pulmonary and Critical Care Medicine, Department of Medicine, Samsung Medical Center, Sungkyunkwan University School of Medicine, 81 Irwon-ro, Gangnam-gu, Seoul, 06351 Republic of Korea; 2 Department of Thoracic and Cardiovascular Surgery, Samsung Medical Center, Sungkyunkwan University School of Medicine, Seoul, Republic of Korea; 3 Patient-Centered Outcomes Research Institute, Samsung Medical Center, Seoul, Republic of Korea; 4 Department of Clinical Research Design and Evaluation, Samsung Advanced Institute for Health Sciences & Technology (SAIHST), Sungkyunkwan University, 81 Irwon-ro, Gangnam-Gu, Seoul, 06351 Republic of Korea

**Keywords:** Chronic obstructive pulmonary disease, Non-small-cell lung cancer, Surgical oncology

## Abstract

Chronic obstructive pulmonary disease (COPD), an established risk factor for lung cancer, remains largely undiagnosed and untreated before lung cancer surgery. We evaluated the effect of perioperative bronchodilator therapy on lung function changes in COPD patients who underwent surgery for non-small cell lung cancer (NSCLC). From a database including NSCLC patients undergoing lung resection, COPD patients were identified and divided into two groups based on the use of bronchodilator during the pre- and post-operative period. Changes in forced expiratory volume in 1 s (FEV_1_) and postoperative complications were compared between patients treated with and without bronchodilators. Among 268 COPD patients, 112 (41.8%) received perioperative bronchodilator, and 75% (84/112) were newly diagnosed with COPD before surgery. Declines in FEV_1_ after surgery were alleviated by perioperative bronchodilator even after adjustments for related confounding factors including surgical extent, surgical approach and preoperative FEV_1_ (adjusted mean difference in FEV_1_ decline [95% CI] between perioperative bronchodilator group and no perioperative bronchodilator group; − 161.1 mL [− 240.2, − 82.0], − 179.2 mL [− 252.1, − 106.3], − 128.8 mL [− 193.2, − 64.4] at 1, 4, and 12 months after surgery, respectively). Prevalence of postoperative complications was similar between two groups. Perioperative bronchodilator therapy was effective to preserve lung function, after surgery for NSCLC in COPD patients. An active diagnosis and treatment of COPD are required for surgical candidates of NSCLC.

## Introduction

Lung cancer is by far the leading cause of cancer deaths worldwide^1^. Chronic obstructive pulmonary disease (COPD) is an independent risk factor for lung cancer development and the most frequent concomitant disease in patients with early stage non-small cell lung cancer (NSCLC)^2,3^. Although surgical resection remains as the cornerstone of curative treatment in early stage NSCLC^4^, patients with COPD are often precluded from lung cancer surgery due to inevitable deteriorations in pulmonary function and the increased risk of postoperative pulmonary complications (PPC) and poor outcome following lung resection^5–7^.

COPD frequently remains under-recognized in both the general population and patients diagnosed with NSCLC^8–10^. In one earlier study, 50.2% of all patients with NSCLC had COPD, while only 7.2% were aware of the disease before their diagnosis of lung cancer^10^. Failure to detect COPD in patients with newly diagnosed lung cancer indicates that these patients have not been treated for COPD and can result in inadequate management as these patients will not benefit from bronchodilator treatments.

Bronchodilators, which significantly improve respiratory symptoms and lung function, are the mainstay of the management of stable COPD^11,12^. A couple of studies have shown that preoperative treatment with bronchodilators significantly increased predicted postoperative pulmonary functions in untreated COPD patients with lung cancer, and some of the patients were eventually able to undergo surgical resection^13–15^. Another study in 20 patients with COPD showed that bronchodilator therapy with tiotropium and salmeterol improved lung function and quality of life at 1 year after surgical resection, compared to a control group^16^. However, these previous studies evaluated fewer than 50 patients. In addition, the recently introduced once-daily dual long-acting bronchodilators, which show greater efficacy to improve lung function^17^, were not adequately evaluated in those studies.

We thus studied the effect of perioperative bronchodilator therapy on postoperative lung function during short-term and long-term periods in patients with COPD who underwent surgical resection for NSCLC. We further compared the development of PPCs between patients who were not treated with bronchodilator and those who were treated with bronchodilator.

## Results

The mean (SD) age of study participants was 66.7 (7.3) years, and the prevalence of perioperative bronchodilator therapy was 41.8% (N = 112); 75% of patients (84/112) were newly diagnosed with COPD and had begun bronchodilator therapy ahead of their lung cancer surgery. Compared to participants who were treated without a bronchodilator perioperatively, those treated with perioperative bronchodilators were more likely to be older (65.8 vs. 68.1, *P* = 0.01), to have squamous cell carcinomas (33.3% vs. 46.4%, *P* < 0.01), and to have lower forced expiratory volume in 1 s (FEV_1_) (2,077 mL vs. 1,851 mL, *P* < 0.01) and % pred FEV_1_ (68.0% vs. 63.2%, *P* < 0.01) (Table 1).

**Table 1:** Characteristics of all study participants according to perioperative bronchodilator use.

	Overall	Perioperative Bronchodilator		
	(N = 268)	No (N = 156)	Yes (N = 112)*	*P* value
**Age (years)**	66.7 (7.3)	65.8 (7.3)	68.1 (7.0)	0.01
**Sex**				0.92
Female	39 (14.6)	23 (14.7)	16 (14.3)	
Male	229 (85.4)	133 (85.3)	96 (85.7)	
**BMI (kg/m**^**2**^**)**				0.97
Underweight	8 (3.0)	5 (3.2)	3 (2.7)	
Normal	87 (32.5)	52 (33.3)	35 (31.3)	
Overweight	67 (25.0)	39 (25.0)	28 (25.0)	
Obese	106 (39.6)	60 (38.5)	46 (41.1)	
**Smoking status**				0.76
Never	32 (11.9)	19 (12.2)	13 (11.6)	
Current smoker	115 (42.9)	64 (41)	51 (45.5)	
Ex-smoker	121 (45.1)	73 (46.8)	48 (42.9)	
**Pulmonary function test**				
FVC (mL)	3,429.5 (746.3)	3,495.1 (745.4)	3,338.1 (741.2)	0.09
FVC, % predicted	82.6 (11.8)	83.5 (11.8)	81.4 (11.7)	0.15
FEV_1_ (mL)	1,982.3 (503.1)	2,076.6 (489.8)	1,851.0 (494)	< 0.01
FEV_1_, % predicted	66 (11.1)	68 (10.2)	63.2 (11.7)	< 0.01
FEV_1_/FVC (%)	58 (8.4)	59.6 (7.6)	55.7 (9.0)	< 0.01
DLco, % predicted	78.0 (16.2)	79.9 (15.4)	75.4 (17.0)	0.024
**Histology**				< 0.01
Adenocarcinoma	146 (54.5)	98 (62.8)	48 (42.9)	
Squamous cell	104 (38.8)	52 (33.3)	52 (46.4)	
Large cell	9 (3.4)	4 (2.6)	5 (4.5)	
Others	9 (3.4)	2 (1.3)	7 (6.3)	
**Location**				0.10
Right upper lobe	76 (28.4)	48 (30.8)	28 (25.0)	
Right middle lobe	11 (4.1)	6 (3.8)	5 (4.5)	
Right lower lobe	49 (18.3)	22 (14.1)	27 (24.1)	
Left upper lobe	87 (32.5)	48 (30.8)	39 (34.8)	
Left lower lobe	45 (16.8)	32 (20.5)	13 (11.6)	
**Type of surgery**				0.35
Wedge resection/segmentectomy	54 (20.1)	27 (17.3)	27 (24.1)	
Lobectomy	194 (72.4)	116 (74.4)	78 (69.6)	
Bilobectomy/pneumonectomy	20 (7.5)	13 (8.3)	7 (6.3)	
**VATS**	163 (60.8)	100 (64.1)	63 (56.3)	0.19
**Clinical stage**				0.17
I	157 (58.6)	94 (60.3)	63 (56.3)	
II	69 (25.7)	43 (27.6)	26 (23.2)	
III	42 (15.7)	19 (12.2)	23 (20.5)	
**Adjuvant treatment**	63 (23.5)	34 (21.8)	29 (25.9)	0.44

Values in the table represent means (standard deviation), or numbers (percent).

*BMI* body mass index, *FEV*_*1*_ forced expiratory volume in 1, *FVC* forced expiratory vital capacity, *VATS* video-assisted thoracic surgery.

*Among 112 patients who used a bronchodilator, 17 (15.2%), 19 (17.0%), 61 (54.5%), and 15 (13.4%) received LAMA, ICS/LABA, LAMA/LABA, and ICS/LAMA/LABA, respectively. There was no ICS monotherapy.

**Figure 1:**
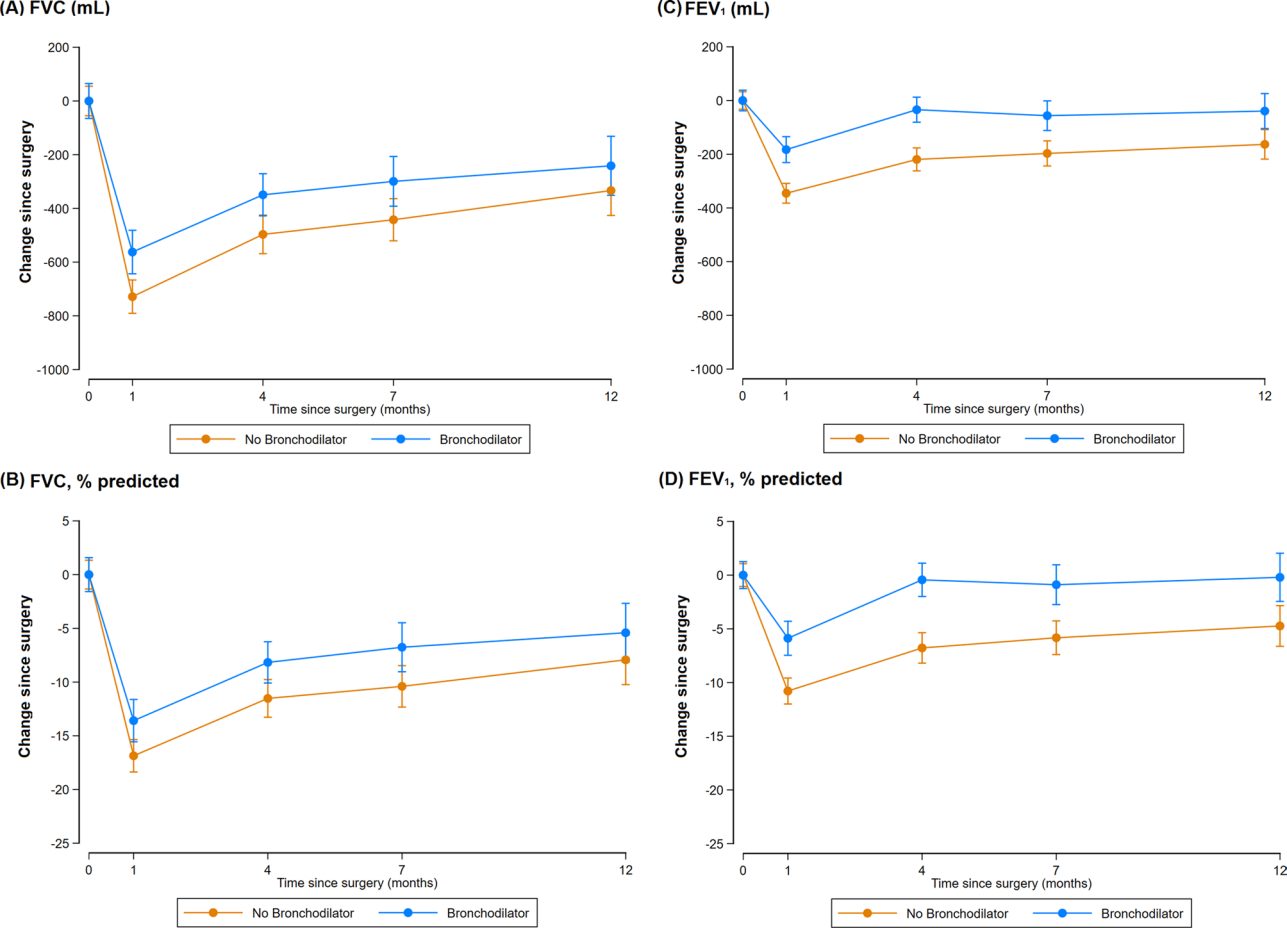
Changes in pulmonary function from baseline to 1, 4, 7, and 12 months following lung resection according to perioperative bronchodilator use.

During the follow-up (the average duration of the follow-up was 10.7 months), a greater decline in FEV_1_ [95% confidence interval (CI)] was observed in patients not treated with perioperative bronchodilator compared with those who received perioperative bronchodilator at 1 month [− 161.1 mL (− 240.2, − 82.0); *P* < 0.001], 4 months [− 179.2 mL (− 252.1, − 106.3); *P* < 0.001], and 12 months [− 128.8 mL (− 193.2, − 64.4); *P* < 0.001] after surgery, respectively (Table 2). When we assessed % pred FEV_1_, participants treated without a perioperative bronchodilator showed larger decline of − 5.5% (− 8.0, − 2.9), − 6.1% (− 8.6, − 3.7), and − 4.6% (− 6.9, − 2.3) in % pred FEV_1_ (95% CI) at 1, 4, and 12 months after surgery, respectively than participants treated with perioperative bronchodilators (Table 2 and Fig. 1). When we conducted inverse probability of treatment weighting (IPTW) of preoperative baseline FEV_1_, the results were similar (data not shown). In terms of forced vital capacity (FVC), patients treated without a perioperative bronchodilator showed greater decline in FVC (95% CI) than those treated with perioperative bronchodilators at 1 months [− 172.6 mL (− 295.1, − 50.1); *P* = 0.006], 4 months [− 128.8 mL (− 250, − 7.6); *P* = 0.037], and 12 months [− 105.4 mL (− 220, 9.1); *P* = 0.071] after surgery, respectively. While the patterns observed for FVC and % pred FVC were similar to those observed for FEV_1_, the differences between the groups treated with and without perioperative bronchodilators were reduced at 12 months after surgery.

**Table 2:** Changes in pulmonary function from baseline to 1, 4, and 12 months following lung resection according to perioperative bronchodilator use.

	No perioperative bronchodilator (N = 156)	Perioperative bronchodilator (N = 112)	Decline in no perioperative bronchodilator group – Decline in perioperative bronchodilator group	
			Difference* between two groups	*P* values*
**FVC (mL)**				
Change from baseline to 1 months after surgery	− 741.5 (− 816.5, − 666.5)	− 568.9 (− 665.8, − 472.1)	− 172.6 (− 295.1, − 50.1)	0.006
Change from baseline to 4 months after surgery	− 485.6 (− 567.5, − 403.7)	− 356.8 (− 446.1, − 267.5)	− 128.8 (− 250.0, − 7.6)	0.037
Change from baseline to 12 months after surgery	− 321.4 (− 394.9, − 248.0)	− 216 (− 303.8, − 128.1)	− 105.4 (− 220.0, 9.1)	0.071
**FVC, % predicted**				
Change from baseline to 1 months after surgery	− 17.2 (− 19.1, − 15.4)	− 13.4 (− 15.8, − 11.0)	− 3.9 (− 6.9, − 0.9)	0.012
Change from baseline to 4 months after surgery	− 11.3 (− 13.4, − 9.3)	− 8.4 (− 10.6, − 6.2)	− 2.9 (− 5.9, 0.1)	0.054
Change from baseline to 12 months after surgery	− 7.6 (− 9.4, − 5.8)	− 4.8 (− 7.0, − 2.6)	− 2.8 (− 5.6, 0)	0.053
**FEV**_**1**_** (mL)**				
Change from baseline to 1 months after surgery	− 345.7 (− 394.0, − 297.5)	− 184.7 (− 247.4, − 122.0)	− 161.1 (− 240.2, − 82.0)	< 0.001
Change from baseline to 4 months after surgery	− 219.9 (− 269.2, − 170.6)	− 40.7 (− 94.3, 13.0)	− 179.2 (− 252.1, − 106.3)	< 0.001
Change from baseline to 12 months after surgery	− 154.5 (− 195.9, − 113.2)	− 25.7 (− 75.1, 23.7)	− 128.8 (− 193.2, − 64.4)	< 0.001
**FEV**_**1**_**, % predicted**				
Change from baseline to 1 months after surgery	− 10.9 (− 12.5, − 9.3)	− 5.5 (− 7.5, − 3.4)	− 5.5 (− 8.0, − 2.9)	< 0.001
Change from baseline to 4 months after surgery	− 6.8 (− 8.4, − 5.1)	− 0.7 (− 2.5, 1.1)	− 6.1 (− 8.6, − 3.7)	< 0.001
Change from baseline to 12 months after surgery	− 4.4 (− 5.9, − 2.9)	0.2 (− 1.6, 2.0)	− 4.6 (− 6.9, − 2.3)	< 0.001

Adjusted for age, sex, body mass index (underweight, normal, overweight, or obese), smoking status (never, past, or current), surgical extent (limited resection, lobectomy, bilobectomy, or pneumonectomy), VATS, and preoperative baseline FEV_1_ (mL).

*Differences in pulmonary function changes between the “no perioperative bronchodilator” group and the “perioperative bronchodilator” group.

When we evaluated if the association between perioperative use of bronchodilator and the change in FEV_1_ at 4 months after surgery differed in pre-specified subgroups, the positive effects of perioperative bronchodilator use were consistent in all subgroups analyzed (all *P*-values for interaction > 0.05; Supplementary Fig. 1).

In the sensitivity analysis in patients with lobectomy (N = 194), those treated without a perioperative bronchodilator showed larger decline in FEV_1_ (95% CI) than those treated with perioperative bronchodilators at 1 month [− 163.0 mL (− 252.5, − 73.5); *P* < 0.001], 4 months [− 155.1 mL (− 241.1, − 68.9); *P* < 0.001], and 12 months [− 130.3 mL (− 206.7, − 53.8); *P* = 0.001] after surgery, respectively (Supplementary Table 1 and Supplementary Fig. 2).

Among 112 patients who used a bronchodilator, 17 (15.2%), 19 (17.0%), 61 (54.5%), and 15 (13.4%) received long-acting muscarinic antagonist (LAMA), inhaled corticosteroid (ICS)/long-acting beta-2 agonist (LABA), LAMA/LABA, and ICS/LAMA/LABA, respectively. There was no ICS monotherapy. For subgroup analysis based on bronchodilator therapy, ICS was not regarded as a bronchodilator therapy. Thus, 36 (32.1%) including ICS/LABA were categorized into mono bronchodilator therapy with either LAMA or LABA, and 76 (67.9%) including ICS/LAMA/LABA were categorized into dual bronchodilator therapy with both LAMA and LABA. At 4 and 12 months, the dual bronchodilator group showed a trend towards less lung function decline compared with the two other groups (*P* for trend < 0.001) (Table 3).

**Table 3:** Changes in pulmonary function from baseline to 1, 4, and 12 months following lung resection between mono and dual bronchodilators.

	No perioperative bronchodilator (N = 156)	Mono bronchodilator (N = 36)	Dual bronchodilator (N = 76)	*P* for trend
**FVC (mL)**				
Change from baseline to 1 months after surgery	− 741.5 (− 816.5, − 666.5)	− 554.3 (− 739.2, − 369.5)	− 568.6 (− 682.1, − 455.1)	0.008
Change from baseline to 4 months after surgery	− 485.6 (− 567.5, − 403.7)	− 399.6 (− 551.2, − 247.9)	− 336.9 (− 447.1, − 226.6)	0.031
Change from baseline to 12 months after surgery	− 321.4 (− 394.9, − 248.0)	− 281.7 (− 435.1, − 128.3)	− 184.1 (− 290.9, − 77.3)	0.041
**FVC, % predicted**				
Change from baseline to 1 months after surgery	− 17.2 (− 19.1, − 15.4)	− 13.4 (− 17.9, − 8.8)	− 13.3 (− 16.1, − 10.5)	0.014
Change from baseline to 4 months after surgery	− 11.3 (− 13.4, − 9.3)	− 8.9 (− 12.6, − 5.1)	− 8.2 (− 11, − 5.5)	0.063
Change from baseline to 12 months after surgery	− 7.6 (− 9.4, − 5.8)	− 6.1 (− 9.9, − 2.3)	− 4.2 (− 6.8, − 1.5)	0.036
**FEV**_**1**_** (mL)**				
Change from baseline to 1 months after surgery	− 345.7 (− 394.0, − 297.5)	− 148.0 (− 268, − 28.0)	− 195.3 (− 268.7, − 122.0)	< 0.001
Change from baseline to 4 months after surgery	− 219.9 (− 269.2, − 170.6)	− 50.1 (− 141.2, 41.0)	− 36.9 (− 103.4, 29.5)	< 0.001
Change from baseline to 12 months after surgery	− 154.5 (− 195.9, − 113.2)	− 32.9 (− 119.3, 53.6)	− 22.5 (− 82.7, 37.7)	< 0.001
**FEV**_**1**_**, % predicted**				
Change from baseline to 1 months after surgery	− 10.9 (− 12.5, − 9.3)	− 4.4 (− 8.3, − 0.5)	− 5.8 (− 8.2, − 3.4)	< 0.001
Change from baseline to 4 months after surgery	− 6.8 (− 8.4, − 5.1)	− 0.4 (− 3.5, 2.6)	− 0.9 (− 3.1, 1.4)	< 0.001
Change from baseline to 12 months after surgery	− 4.4 (− 5.9, − 2.9)	0.5 (− 2.6, 3.7)	0.1 (− 2.1, 2.2)	< 0.001

ICS was not regarded as a bronchodilator therapy. Thus, 36 (32.1%) including ICS/LABA were categorized into mono bronchodilator therapy with either LAMA or LABA, and 76 (67.9%) including ICS/LAMA/LABA were categorized into dual bronchodilator therapy with both LAMA and LABA.

Adjusted for age, sex, body mass index (underweight, normal, overweight, or obese), smoking status (never, past, or current), surgical extent (limited resection, lobectomy, bilobectomy, or pneumonectomy), VATS, and preoperative baseline FEV_1_ (mL).

The frequency of overall PPC was 15.7% (n = 42), which did not differ by perioperative bronchodilator therapy (16.7% vs. 14.3%, P = 0.597). Among PPCs, acute respiratory distress syndrome (ARDS) or pneumonia was the most frequent (n = 32), followed by bronchoscopic toileting (n = 8) and COPD acute exacerbation (n = 6). Postoperative cardiovascular complication (PCC) developed in 37 (13.8%) patients, of which atrial fibrillation (AF) (n = 35) was the most common. In multivariable analysis, there was no significant difference between two groups in terms of PPC and PCC risk (Table 4). In patients who received perioperative bronchodilators, 16 (14.2%) developed AF, while 19 (12.2%) of those treated without a perioperative bronchodilator developed AF (*P* = 0.064) (data not shown). Hospital length of stay, intensive care unit (ICU) length of stay, and readmission to ICU also did not differ between two groups (Table 4).

**Table 4:** Postoperative complications and hospital length of stay according to perioperative bronchodilator.

	No perioperative bronchodilator (N = 156)	Perioperative Bronchodilator (N = 112)	*P* value
**Postoperative pulmonary complication (PPC)**			
No. of patients (%)	26 (16.7)	16 (14.3)	0.597
Adjusted* OR (95% CI)	Reference	0.58 (0.27, 1.25)	0.162
**Postoperative cardiovascular complication (PCC)**			
No. of patients (%)	20 (12.8)	17 (15.2)	0.581
Adjusted* OR (95% CI)	Reference	1.05 (0.49, 2.27)	0.899
**PPC or PCC**			
No. of patients (%)	37 (23.7)	28 (25.0)	0.809
Adjusted* OR (95% CI)	Reference	0.86 (0.46, 1.60)	0.632
**Hospital length of stay, days**	8.6 (4.4)	9.6 (6.0)	0.120
**ICU length of stay, days**	1.2 (1.1)	1.2 (0.9)	0.926
**ICU readmission during hospitalization for surgery, n (%)**	4 (2.6)	5 (4.5)	0.498

Values in the table are n (percent), mean (standard deviation) or adjusted odds ratio (95% confidence interval).

*Adjusted for age, sex, body mass index (underweight, normal, overweight, or obese), smoking status (never, past, or current), surgical extent (limited resection, lobectomy, bilobectomy, or pneumonectomy), VATS, preoperative baseline FEV_1_ (mL) and DLco (% predicted).

## Discussion

In this study in patients with COPD who underwent lung cancer surgery, we found that only 41.8% of patients were treated with bronchodilators during the perioperative period, and that most of these patients (75%) were diagnosed with COPD and started the bronchodilator therapy ahead of their lung cancer surgery. Importantly, our study demonstrates that declines in lung function following surgery, especially declines in FEV_1_, were alleviated by the use of perioperative bronchodilators at all time points, including 1, 4, and 12 months postoperatively. This finding persisted after the analyses were adjusted for age, sex, BMI, smoking status, surgical extent, surgical approach and preoperative baseline FEV_1_. In addition, the positive effects of perioperative use of bronchodilator were consistent in all subgroups analyzed. To our knowledge, this is the largest study investigating the usefulness of perioperative bronchodilator therapy in patients with lung cancer with COPD.

COPD subliminally coexists with lung cancer. A previous study showed a prevalence of coexisting COPD in lung cancer of 50%, six times greater prevalence of COPD in newly diagnosed lung cancer cases than in an age-, sex-, and smoking-matched control group^18^. Another study also showed that COPD was present in 50.2% of smokers diagnosed with NSCLC, and most of the patients (92.8%) with COPD were unaware of the disease before their diagnosis of lung cancer^10^. In line with these findings, only 42% of the patients with COPD in our study were treated with bronchodilators during the perioperative period, and most patients began bronchodilator therapy with their COPD diagnosis during the preparation for the lung cancer surgery. These findings indicate that the underdiagnosis and undertreatment of COPD among patients with NSCLC remain common in the clinical practice.

Generally, our findings show that lung function dramatically dropped at 1 month after surgery, slowly recovered until postoperative month 4, and was stable by postoperative month 12. Our findings also show that the perioperative management of COPD using bronchodilators significantly mitigated the lung function decline from baseline, particularly the decline in FEV_1_, compared to patients who were not treated with perioperative bronchodilator. Numerically, the differences in FEV_1_ reductions from baseline between the two groups are greater than 100 mL, which corresponds to the minimal clinically important differences in FEV_1_ in COPD^19^. Furthermore, in some patients who began bronchodilator treatment just before surgical resection, FEV_1_ recovered after resection, up to the baseline FEV_1_ before the initiation of bronchodilator treatment, which was not observed in patients not treated with perioperative bronchodilator (Fig. 1). This significant alleviation of postoperative lung function decline may reduce symptom burden such as dyspnoea caused by lung resection. The detection and proper management of COPD is thus necessary during the preparation for surgical treatment in patients with newly diagnosed lung cancer.

For some patients with severe bullous emphysema, lung resection (especially upper lobectomy) might have lung volume reduction effect^20^. In this regard, we evaluated the emphysema index (EI) in preoperative chest computed tomography (CT) scan of 120 patients who underwent upper lobectomy, using software (Synapse 3D Vincent, Fuji Film, Tokyo, Japan). After excluding four patients with poor-quality image, EI was measured in 116 patients. The number of patients with EI of 5% or more, which is considered as a threshold for clinical importance^21^, was only 15 (13%), and it did not differ between groups with and without bronchodilator therapy (11.9% vs. 14.3%, *P* = 0.710). Thus, we think that the difference of lung function decline between two groups is unlikely due to a different effect from lung volume reduction.

Among the different bronchodilators, dual bronchodilators showed favourable outcomes in terms of mean changes in FEV_1_ from baseline, compared to mono bronchodilators that did not show statistically significant changes. The recently introduced dual once-daily bronchodilators have been shown to have greater efficacy for lung function improvement with comparable safety profiles over monocomponent^17^. Given the similar price range for dual and mono bronchodilators in South Korea, dual once-daily bronchodilators might offer more benefits for the preservation of lung function after surgical resection.

Poor pulmonary function is the major barrier for COPD patients to receive pulmonary resection due to the heightened risk of PPC, which is a major cause of postoperative morbidity and mortality^5,22^. Decline in lung volumes with atelectasis from the combined effects of supine position, general anesthesia, thoracic incision, and respiratory muscles or diaphragmatic dysfunction, results in the development of PPC^23,24^. In this study with COPD patients at least moderate degree of airflow limitation (FEV_1_ < 80% pred), the overall risk of PPC was 15.7%. Of note, PPC prevalence did not differ by perioperative bronchodilator therapy. However, compared to non-perioperative bronchodilator group, those in perioperative bronchodilator group were older and had lower baseline FEV_1_ and DLco, which are well known risk factors for PPC^22,25^. Thus, similar prevalence in PPC between two groups could be a signal of possible benefit of perioperative bronchodilator in preventing PPCs, which must be confirmed in future study.

There is potential cardiovascular safety concerns related to long-acting bronchodilator therapy, especially tachyarrhythmias occurring during the postoperative period^26,27^. In our study, 35 (13.1%) patients developed postoperative AF, with no difference according to perioperative bronchodilator use. In addition, we observed only one case of non-ST-segment elevation myocardial infarction in the non-perioperative bronchodilator group.

This study has several limitations. Firstly, it was performed at a single tertiary hospital and represents a retrospective analysis. There could be unmeasured confounders that might have led to the prescription of bronchodilators. Also, we excluded patients who discontinued bronchodilator after surgery (N = 19) and those who started bronchodilator after surgery (N = 18). This per-protocol analysis is subject to confounding. Thus, future studies with prospective designs are required to validate our findings. Secondly, COPD was defined based on pre-bronchodilator pulmonary function tests, and we may have misclassified some patients^28^. Thus, we limited subjects to patients with FEV_1_ < 80% as well as FEV_1_/FVC < 70%, as these have a high probability of having COPD. Most patients in the non-bronchodilator group did not undergo post-bronchodilator spirometry, which reflects a real-world clinical setting. Given the benefit that bronchodilator therapy showed in our study, active screening for COPD including post-bronchodilator spirometry should be part of preoperative evaluations of patients with lung cancer. Thirdly, not all patients underwent repeated pulmonary function tests after the initiation of bronchodilator therapy before surgical resection. Thus, preoperative responsiveness to inhalers including dual long-acting bronchodilators could not be assessed. Instead, we used the results of baseline lung function tests that were obtained before the initiation of bronchodilator therapy in patients who just began the treatment. Considering that the majority of our patients with COPD newly began the bronchodilator treatment, we assumed that reduced postoperative lung function decline observed in the group treated with perioperative bronchodilators might be attributable to a significant increase in FEV_1_ before surgical resection, as a combined effect of newly prescribed bronchodilator treatment and smoking cessation, based on previous studies^13,15^. In addition, we did not have data on the compliance or inhaling techniques applied during bronchodilator therapy, but we assumed that compliance rates were good before surgical resection, leading to greater benefits. Finally, information regarding respiratory symptoms, quality of life, or physical activities was not uniformly available. We can therefore not be certain that the beneficial effect of bronchodilator use on postoperative lung functions is accompanied by improvements in the patients’ symptoms or quality of life. Indeed, no specific guideline for physical activity and rehabilitation prior to surgery are available at this time. Future studies therefore need to assess comprehensive care approaches including proper treatment of COPD as well as rehabilitation.

In conclusion, perioperative treatment with bronchodilators in patients with COPD shows benefits in the alleviation of the reduction in lung function, in particular FEV_1_, after pulmonary resection for NSCLC. An active diagnosis of COPD and treatment with bronchodilators are thus needed for patients with NSCLC scheduled to undergo surgical resection.

## Methods

### Study subjects

This was a retrospective cohort study in patients with NSCLC who underwent curative intent surgical resection at Samsung Medical Center, Seoul, South Korea, between January 2016 and December 2018. Since our objective was to evaluate the effect of perioperative bronchodilator therapy and the lung function decline following surgery in NSCLC patients with COPD, we identified 664 subjects who had COPD (defined as pre-bronchodilator FEV_1_/FVC < 0.70 and FEV_1_ < 80% pred) at the time of their lung cancer diagnosis.

**Figure 2:**
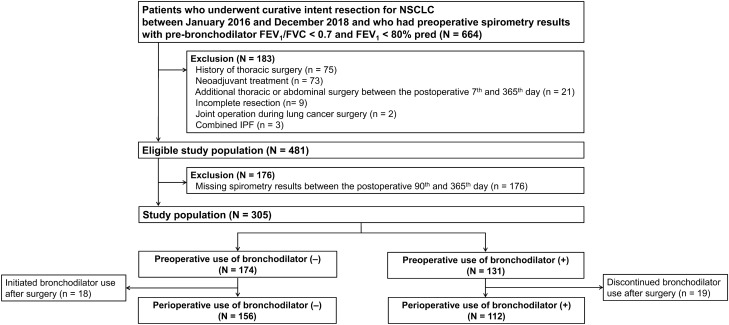
Flow chart.

We excluded patients who had previously undergone thoracic surgery (n = 75), neoadjuvant treatment (n = 73), additional thoracic or abdominal surgery between the 7th and 365th postoperative day (n = 21), incomplete resection (n = 9), or joint operation during lung cancer surgery (n = 2), and those who had combined idiopathic pulmonary fibrosis (n = 3). Of the eligible patients, 176 patients whose records did not contain spirometry test data acquired between the 90th and 365th postoperative day were excluded. We then also excluded patients who discontinued bronchodilator treatment after surgery (n = 19) and those who began bronchodilator treatment only after surgery (n = 18). The final sample consisted of 268 patients (229 men and 39 women, Fig. 2).

The Institutional Review Board of the Samsung Medical Center approved this study (IRB No. 2020-01-131-001) and waived the requirement for informed consent, as only de-identified data routinely collected during clinic visits were analysed. This study was performed in accordance with relevant guidelines/regulations of our institutions.

### Data collection

Demographics, clinical data, and treatment details were obtained from electronic medical records. Demographic information at diagnosis included age, sex, body mass index, and smoking status. Clinical information included pathologic stages, based on the American Joint Committee on Cancer, 7th Edition ^29^, histology (adenocarcinoma, squamous cell carcinoma, large cell carcinoma, and others), largest tumor size, and lobar location. Treatment information included extent of lung resection (limited resection, lobectomy, bilobectomy, or pneumonectomy), use of video-assisted thoracic surgery (VATS), and adjuvant treatment.

### Bronchodilator therapy

In this study, bronchodilator therapy was confined to LAMA, LABA, or LAMA/LABA with or without ICS. ICS administration was not counted as bronchodilator therapy. Perioperative use of a bronchodilator was defined as at least one prescription of aforementioned inhalers both (1) within 60 days prior to the surgery and (2) within 180 days after the surgery. Patients who underwent preoperative bronchodilator therapy were further categorised into a monotherapy (either LAMA or LABA) and a dual therapy (LAMA/LABA) group, irrespective of ICS use.

### Pulmonary function tests

Preoperative spirometry was generally performed within 1 or 2 months prior to surgery, and postoperative spirometry was performed during follow-up, at around the 1st (0–2nd), 4th (3–5th), 7th (6–8th), and 12th (9–15th) months. All spirometry tests were performed in a pulmonary function lab, using a Vmax 22 system (SensorMedics, Yorba Linda, CA, USA) according to the American Thoracic Society/European Respiratory Society criteria^30^. Absolute FEV_1_ and FVC values were obtained and the percentages of predicted FEV_1_ and FVC were calculated using a reference equation obtained from a representative South Korean sample^31^. The diffusing capacity of carbon monoxide of the lung (DLco) was also routinely measured preoperatively, using the same apparatus, and absolute DLco values (mL/mmHg/min) were converted to percentages of predicted values using a formula based on a representative South Korean sample^32,33^.

### Postoperative cardiopulmonary complications

PPC and PCC occurred during hospitalization or readmission within the first 60 days postoperatively were identified from prospectively collected data. PPC was defined as any of the following condition; (1) significant atelectasis requiring bronchoscopic toileting or reintubation; (2) pneumonia; (3) ARDS, (4) acute exacerbation of COPD^24,34^. PCC was defined as following; (1) acute myocardial infarction; (2) atrial arrhythmia associated with the use of antiarrhythmic drugs or anticoagulant; (3) ventricular tachycardia/fibrillation. (4) cardiac arrest or any cardiac related death^34,35^. All postoperative complications were classified according to Clavien–Dindo classification^36^, grade II or higher complication were included for analysis.

### Statistical analysis

Differences in baseline characteristics were compared between patient groups treated with and without the perioperative use of a bronchodilator using χ^2^ tests for categorical variables and t-tests for continuous variables. The primary outcome was the change in FEV_1_ following perioperative bronchodilator treatment. We compared quantitative changes in pulmonary function, including absolute values of FVC and FEV_1_, and the percentages of predicted values (% pred) for FEV_1_ and FVC at each time point in patient groups treated with and without a perioperative bronchodilator using linear mixed models with random intercepts and random slopes^37^. We estimated differences in the changes of pulmonary function from preoperative values (with 95% CI) between participants treated with and those treated without a perioperative bronchodilator. To control for potential confounding factors, we adjusted analyses for age, sex, body mass index [underweight (< 18.5 kg/m^2^), normal (18.5–22.9 kg/m^2^), overweight (23–24.9 kg/m^2^), or obese (≥ 25 kg/m^2^)], smoking status (never, past, or current), surgical extent (limited resection, lobectomy, bilobectomy, or pneumonectomy), VATS, and preoperative baseline FEV_1_ (mL). We also performed the same analysis using IPTW of preoperative baseline FEV_1_^38^. To evaluate if changes in pulmonary function correlated with the type of bronchodilator used, we conducted additional analyses after separating patients into mono and dual bronchodilator groups. In addition, we performed stratified analyses to evaluate if the association of perioperative use of bronchodilator with change in FEV_1_ at 4 months after surgery differed in pre-specified subgroups defined by age (< 65 vs. ≥ 65 years), sex, obesity (no vs. yes), smoking (never vs. ever), and adjuvant therapy (no vs. yes). We also performed sensitivity analyses in patients who received lobectomy (N = 194). We considered a *P*-value < 0.05 as statistically significant. All analyses were performed using STATA version 15 (StataCorp LP, College Station, TX, USA).

## Supplementary Information

Supplementary Table 1.

Supplementary Figure 1.

Supplementary Figure 2.
