# Supplementary material for: Effect of perioperative bronchodilator therapy on postoperative pulmonary function among lung cancer patients with COPD
Source: Sci Rep. 2021 Apr 16;11:8359. doi: 10.1038/s41598-021-86791-1 (PMC8052420; doi:10.1038/s41598-021-86791-1)
Supplement: Supplementary file 1 — Supplementary Table 1. [file 41598_2021_86791_MOESM1_ESM.docx]

**Effect of Perioperative Bronchodilator Therapy on Postoperative Pulmonary Function among Lung Cancer Patients with COPD**

Sun Hye Shin^1*^, Sumin Shin^2*^, Yunjoo Im^1*^, Genehee Lee^3^, Byeong-Ho Jeong^1^, Kyungjong Lee^1^, Sang-Won Um^1^, Hojoong Kim^1^, O Jung Kwon^1^, Jong Ho Cho^2^, Hong Kwan Kim^2^, Yong Soo Choi^2^, Jhingook Kim^2^, Jae Ill Zo^2^, Young Mog Shim^2^, Juhee Cho^4^, Danbee Kang^4†^, Hye Yun Park^1†^

**Supplementary Figure Legends**

**Supplementary Figure 1. Difference in FEV_1_ (mL) changes from baseline to the postoperative 4 months in pre-specified subgroups**

**Supplementary Figure 2. Changes in pulmonary function from baseline to 1, 4, 7, and 12 months following lung resection according to perioperative bronchodilator use in patients received lobectomy (N = 194)**

**Supplement table 1. Changes in pulmonary function from baseline to 1, 4, and 12 months following lung resection according to perioperative bronchodilator among patients with lobectomy (N = 194)**

|  |  | **No Perioperative Bronchodilator**  **(N = 116)** | **Perioperative Bronchodilator**  **(N = 78)** | **Decline in no perioperative bronchodilator group**  **– Decline in perioperative bronchodilator group** | |
| --- | --- | --- | --- | --- | --- |
|  |  |  |  | **Difference* between**  **two groups** | ***P* values*** |
| **FVC (mL)** | |  |  |  |  |
|  | Change from baseline to 1 months after surgery | -777.0 (-859.1, -695.0) | -668.9 (-780.4, -557.3) | -108.2 (-246.7, 30.3) | 0.126 |
|  | Change from baseline to 4 months after surgery | -481.8 (-569.9, -393.6) | -440 (-540.7, -339.2) | -41.8 (-175.7, 92.1) | 0.54 |
|  | Change from baseline to 12 months after surgery | -302.1 (-383.3, -221) | -237.2 (-338.7, -135.8) | -64.9 (-194.8, 65.0) | 0.328 |
| **FVC, % predicted** | |  |  |  |  |
|  | Change from baseline to 1 months after surgery | -18.0 (-20.0, -16.0) | -15.8 (-18.5, -13.0) | -2.2 (-5.6, 1.2) | 0.207 |
|  | Change from baseline to 4 months after surgery | -11.1 (-13.3, -9.0) | -10.4 (-12.9, -7.9) | -0.8 (-4.1, 2.5) | 0.644 |
|  | Change from baseline to 12 months after surgery | -6.9 (-8.9, -5.0) | -5.2 (-7.7, -2.7) | -1.8 (-5.0, 1.4) | 0.281 |
| **FEV_1_ (mL)** | |  |  |  |  |
|  | Change from baseline to 1 months after surgery | -382.8 (-435.7, -329.9) | -219.8 (-292, -147.6) | -163.0 (-252.5, -73.5) | < 0.001 |
|  | Change from baseline to 4 months after surgery | -222.2 (-279.1, -165.3) | -67.1 (-131.9, -2.3) | -155.1 (-241.3, -68.9) | < 0.001 |
|  | Change from baseline to 12 months after surgery | -159.8 (-207.6, -112.0) | -29.5 (-89.2, 30.2) | -130.3 (-206.7, -53.8) | 0.001 |
| **FEV_1_, % predicted** | |  |  |  |  |
|  | Change from baseline to 1 months after surgery | -12.1 (-13.9, -10.3) | -6.6 (-9.0, -4.2) | -5.5 (-8.5, -2.5) | < 0.001 |
|  | Change from baseline to 4 months after surgery | -6.8 (-8.7, -4.9) | -1.6 (-3.8, 0.5) | -5.2 (-8.1, -2.3) | < 0.001 |
|  | Change from baseline to 12 months after surgery | -4.4 (-6.1, -2.6) | 0.2 (-2.0, 2.3) | -4.5 (-7.3, -1.8) | 0.001 |

^*^ Differences in pulmonary function changes between the “no perioperative bronchodilator” group and the “perioperative bronchodilator” group.

Adjusted for age, sex, body mass index (underweight, normal, overweight, or obese), smoking status (never, past, or current), VATS, and pre-operative baseline FEV_1_ (mL).
