# Supplementary figures and images for: Effect of perioperative bronchodilator therapy on postoperative pulmonary function among lung cancer patients with COPD
Source: Sci Rep. 2021 Apr 16;11:8359. doi: 10.1038/s41598-021-86791-1 (PMC8052420; doi:10.1038/s41598-021-86791-1)

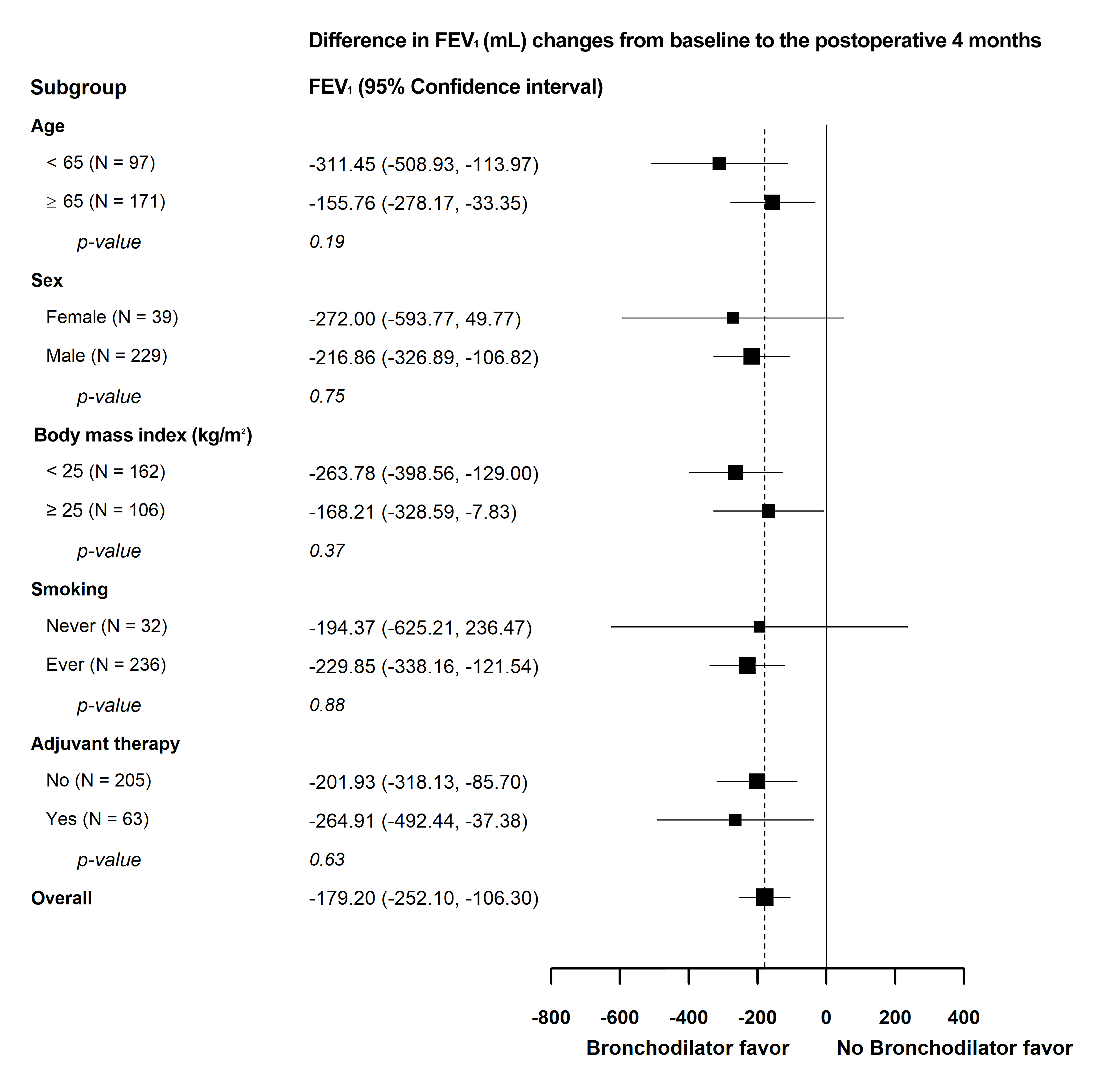

Supplement: Supplementary file 2 — Supplementary Figure 1. [file 41598_2021_86791_MOESM2_ESM.tif]

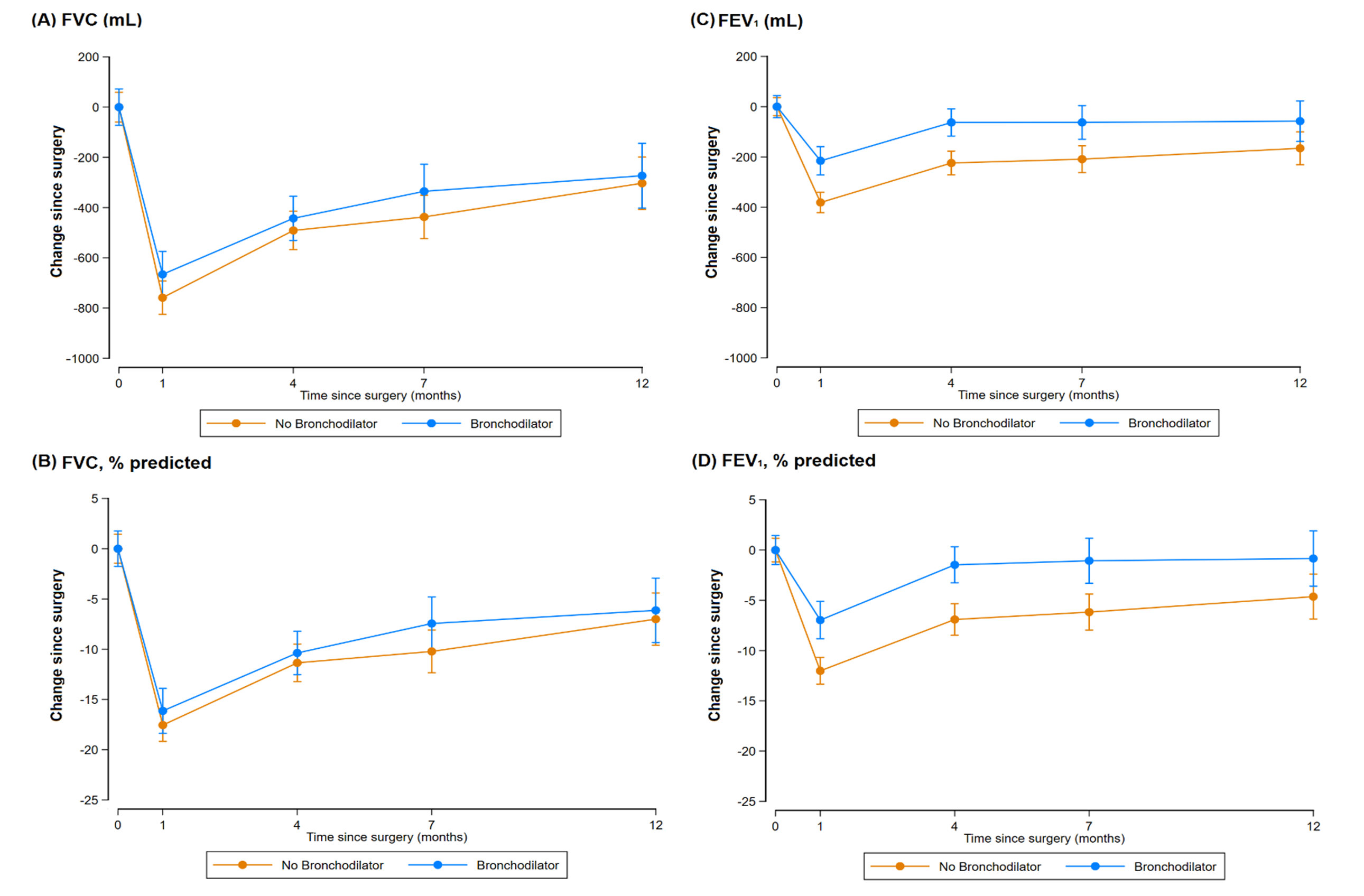

Supplement: Supplementary file 3 — Supplementary Figure 2. [file 41598_2021_86791_MOESM3_ESM.tif]
